# Comprehensive phytochemical and toxicological analysis of *Chenopodium ambrosioides* (L.) fractions

**DOI:** 10.1515/biol-2022-0895

**Published:** 2024-06-26

**Authors:** Soufiane Drioua, Mouna Ameggouz, Amine Assouguem, Mohammed Kara, Riaz Ullah, Ahmed Bari, Rachid Lahlali, Hafize Fidan, Otman El-Guourrami, Fatima Zahra Benkhouili, Yagoubi Maamar, Hanane Benzeid, Anass Doukkali

**Affiliations:** Laboratory of Analytical Chemistry, Department of Drug Sciences, Faculty of Medicine and Pharmacy, Mohammed V University in Rabat, Rabat, Morocco; Laboratory of Functional Ecology and Environment, Faculty of Sciences and Technology, Sidi Mohamed Ben Abdellah University, PO. box 2202 Imouzzer Street, Fez, 30000, Morocco; Department of Plant Protection and Environment, National School of Agriculture, Meknes, Morocco; Laboratory of Biotechnology, Conservation and Valorisation of Natural Resources (LBCVNR), Faculty of Sciences Dhar El Mehraz, Sidi Mohamed Ben Abdallah University, BP 1796 Atlas, Fez, 30000, Morocco; Department of Pharmacognosy, College of Pharmacy, King Saud University, Riyadh, Saudi Arabia; Department of Pharmaceutical Chemistry, College of Pharmacy, King Saud University, Riyadh, Riyadh Province 11451, Saudi Arabia; Department of Tourism and Culinary Management, Faculty of Economics, University of Food Technologies, Plovdiv, Bulgaria; Laboratory of Medicinal Chemistry, Department of Drug Sciences, Faculty of Medicine and Pharmacy, Mohammed V University in Rabat, Rabat, Morocco; Laboratory of Microbiology, Clinical Biology Department, Faculty of Medicine and Pharmacy, Mohammed V University in Rabat, Rabat, Morocco

**Keywords:** *Chenopodium ambrosioides*, LC–MS/MS, phytochemical, toxicity

## Abstract

*Chenopodium ambrosioides* aerial parts have been historically employed in traditional medicine for addressing various ailments such as headaches, abdominal discomfort, joint issues, and respiratory disorders, alongside treatments for lice and warts. This study aimed to conduct a comprehensive phytochemical analysis of *C. ambrosioides* and assess the acute and subacute toxicity of oral treatments using fractions in preclinical trials. Spectrophotometric analysis via LC–MS/MS was used to characterize the plant’s chemical composition. Acute toxicity evaluation followed Organisation for Economic Co-operation and Development code 42 guidelines, conducted on adult male and female Wistar strain mice. Subsequently, Swiss mice were divided into six groups for the subacute toxicity study, receiving oral doses of 200 mg/kg extracts and fractions for 28 days. Daily observations and biochemical analyses were performed, with LC–MS/MS revealing a diverse array of compounds including organic acids, flavonoids, phenolic acids, rutin, hesperidin, nicotiflorine, and fumaric acid. Results indicated no lethality or alterations in body weight in treated groups, though some organ weight changes were noted. Biochemical analyses demonstrated values within the normal range for all groups, suggesting that the treatments did not induce adverse effects. Acute and subacute treatments with fractions did not result in lethality or toxic alterations at therapeutic doses, implying the safety of the product at appropriate levels. This study underscores the potential of *C. ambrosioides* as a safe therapeutic option warranting further exploration.

## 1 Introduction

The utilization of medicinal plants as primary healthcare solutions in developing nations is deeply entrenched, primarily due to financial constraints and accessibility issues. This reliance on traditional medicine, underscored by UNESCO’s emphasis on the accessibility and financial limitations of populations in need, has prompted the World Health Organization to advocate for a reevaluation of traditional medicine to better address healthcare needs in resource-limited regions [1,2]. Ethnobotanical studies, particularly prevalent in Africa, have meticulously documented numerous medicinal plant species, with over 5,000 species recognized, including 761 with medicinal properties, and 1,421 distinct medicinal formulations found specifically in Côte d’Ivoire [3,4]. However, despite this resurgence of interest in phytotherapy, there is a noticeable emphasis on therapeutic efficacy over the toxicological aspects within the field’s advancements.

*Chenopodium ambrosioides* L., originating from Central America, has attracted considerable attention for its historical use across various cultures. It has been employed as an anti-helminthic, anti-inflammatory, anti-tumoral, and wound-healing agent, particularly noted for its traditional use in treating *Leishmania*-induced skin ulcers.

While these traditional practices highlight the significant role of medicinal plants in providing healthcare in resource-limited settings, they also underscore the critical need for rigorous scientific inquiry. Despite their historical and cultural significance, there is a noticeable lack of comprehensive toxicological studies on these remedies [5,6]. This gap in understanding has prompted calls for further scientific investigations, particularly in understanding the potential toxicological aspects of these remedies. Such endeavors aim to bridge traditional knowledge with modern scientific rigor, ensuring the safe and effective integration of these medicinal plants into formal healthcare systems [7,8].

Phytochemicals, acknowledged as bioactive compounds, constitute a broad spectrum of plant secondary metabolites comprising numerous natural substances. This encompasses a diverse array of compounds, such as phenols, phenolic acids, flavonoids, tannins, saponins, alkaloids, steroids, steroids, lignins, glycosides, phenylpropanoid glycerols, and isoprene-derived terpenoids (isoprenoids) [9].

Several biological activities of *C. ambrosioides* have been scientifically confirmed, including its anti-helminthic effects against various parasites and anti-inflammatory properties. However, reports of genotoxic effects and fatalities from overdoses highlight the importance of understanding the toxicological mechanisms underlying its usage [10,11,12].

Considering these considerations, our study aims to elucidate the specific biological activities and potential health benefits associated with phytochemicals from *C. ambrosioides* [13]. Through the preparation of four fractions using solvents of increasing polarity and subsequent evaluation of their acute and subacute toxicity following Organisation for Economic Co-operation and Development (OECD) 423 guidelines, we seek to contribute to our understanding of the therapeutic potential of this plant. By assessing its toxicity, we aim to provide valuable insights into its safety profile, thus encouraging further exploration of natural remedies in healthcare [14,15].

Ultimately, the literature review takes the reader from what is known about a subject to the gaps that remain in our understanding, underpinning the study that follows [16]. This research endeavors to shed light on the therapeutic potential of *C. ambrosioides* while also highlighting the importance of understanding its toxicological profile for safe and effective integration into healthcare practices.

## 2 Materials and methods

### 2.1 Plant material

The entirety of the *C. ambrosioides* plant (Chenopodiaceae) was collected between May and July 2021 in the Rabat region of Morocco (Geographical coordinates: 33.970878, −6.814212). Botanical authentication of the plant was conducted by the floristics team at the Scientific Institute of Rabat. This specific specimen has been deposited in the herbarium of the Scientific Institute of Rabat and bears the sample number RAB113708. Samples were dried in the laboratory at room temperature before undergoing extraction. The dried plant material was pulverized using a Binatone Moulinex mixer.

### 2.2 Preparation of fractions

To prepare the fractions, dried aerial parts (50 g) of *C. ambrosioides* underwent Soxhlet extraction using cyclohexane. After dry evaporation, the cyclohexane fraction was obtained. The remaining residue was dried in an oven for 24 h and subjected to hydroalcoholic maceration (ethanol/water: 50/50). Following filtration and ethanol evaporation, successive liquid–liquid separations were performed on the aqueous phase using solvents of increasing polarity (ethyl acetate and n-butanol) and repeated three times for each solvent (100 ml). After dry evaporation, fractions of ethyl acetate and *n*-butanol were obtained.

The extraction rate was calculated using the following formula:

\[R=({M}_{i}/M)\times 100,]\]

where *M*_*i*_ is the mass of the extract and *M* is the mass of the initial plant material.

### 2.3 Conditions of mass spectrometer and chromatography

We utilized an ultra-high-performance liquid chromatography (UHPLC) Shimadzu-Nexera coupled with a tandem mass spectrometer for the quantitative evaluation of 53 phytochemicals. The reverse-phase UHPLC system was equipped with an automatic sampler (SIL-30AC model), a column oven (CTO-10ASvp model), binary pumps (LC-30AD model), and a degasser (DGU-20A3R model).

Chromatographic conditions were optimized to achieve optimal separation of the 53 phytochemicals and overcome suppression effects. Several columns were tested and applied, including the Agilent Poroshell 120 EC-C18 model (150 mm × 2.1 mm, 2.7 µm) and the RP-C18 Inertsil ODS-4 model (100 mm × 2.1 mm, 2 µm). Different mobile phases such as acetonitrile and methanol were used, along with various mobile phase additives such as ammonium formate, formic acid, ammonium acetate, and acetic acid. Column temperatures varied between 25, 30, 35, and 40°C until optimal conditions were attained.

Ultimately, chromatographic separation was performed on an analytical reverse-phase column, Agilent Poroshell 120 EC-C18 (150 mm × 2.1 mm, 2.7 µm). The column temperature was maintained at 40°C. The eluent gradient consisted of eluent A (water + 5 mM ammonium formate + 0.1% formic acid) and eluent B (methanol + 5 mM ammonium formate + 0.1% formic acid). The following gradient elution profile was used: 20–100% B (0–25 min), 100% B (25–35 min), and 20% B (35–45 min). Additionally, the solvent flow rate and injection volume were set at 0.5 mL/min and 5 µL, respectively.

Mass spectrometry detection was conducted using a Shimadzu LCMS-8040 tandem mass spectrometer equipped with an electrospray ionization (ESI) source operating in both negative and positive ionization modes. LC–ESI-MS/MS data were acquired and processed using LabSolutions software (Shimadzu).

The multiple reaction monitoring (MRM) mode was employed for phytochemical quantification. The MRM method was optimized to selectively detect and quantify phytochemical compounds based on screening specific precursor ion transitions of phytochemicals to fragment ions. Collision energies were optimized to generate optimal fragmentation of phytochemicals and maximum transmission of desired product ions.

The MS operating conditions were as follows: drying gas flow rate (*N*2), 15 L/min; nebulizing gas flow rate (*N*2), 3 L/min; desolvation line temperature, 250°C; heating block temperature, 400°C, and interface temperature, 350°C.

These coupled UHPLC–chromatograph mass spectrometer conditions enabled high-quality data acquisition for the quantitative analysis of the 53 phytochemicals, crucial for precise sample evaluation. Fine adjustments of chromatographic and spectrometric parameters optimized method sensitivity and selectivity, ensuring reliable and reproducible results.

### 2.4 Evolution of acute toxicity

The acute toxicity of the studied fractions of *C. ambrosioides* was assessed in adult male and female Wistar strain mice (*Mus musculus*) following OECD guidelines, code 42, weighing between 21 and 36 g. The animals were sourced from the Faculty of Medicine and Pharmacy’s Animal Facility in Rabat, under standard experimental conditions. They were marked twice a week to maintain their identity within each batch. The animals were divided into multiple groups and housed in polypropylene cages with wood shavings, each labeled with batch name, treatment details, and experimentation dates.

For 7 days preceding each experiment, the mice underwent an adaptation period where they had unrestricted access to water ad libitum and standard feed, under controlled light and temperature conditions (12-h light/dark cycle at 27 ± 2°C). The experiments were conducted following internationally accepted guidelines for evaluating the safety and efficacy of herbal-based medicines [17].

### 2.5 Subacute toxicity test

The subacute oral toxicity study was conducted following the guidelines set by the (OECD 407, 2008). Animals were randomly divided into six groups, each consisting of ten animals. Group I, designated as the control group, received distilled water (vehicle) orally for a duration of 28 days. Groups II, III, IV, and V received a daily oral dose of 200 mg/kg of the fractions for a continuous 28-day period. The body weight of the subjects was recorded weekly throughout the study period.

#### 2.5.1 Determination of biochemical parameters:

Following the experimental period, blood samples were collected from the caudal vein using heparinized tubes for hematological studies. Non-heparinized tubes were used to obtain serum through centrifugation at 3,000 rpm for 10 min, intended for subsequent biochemical analyses.

Hematological parameters, including total hemoglobin, red blood cell count, white blood cell count, and platelet count, were determined using a fully automated analyzer (Architect c8000, Clinical Chemistry System, Chicago, IL, USA).

Simultaneously, serum levels of aspartate aminotransferase (AST), alanine aminotransferase (ALT), urea (UR), creatinine (CR), cholesterol, triacylglycerols, high-density lipoproteins, and low-density lipoproteins (LDL) were precisely determined.

## 3 Results

**Figure 1:**
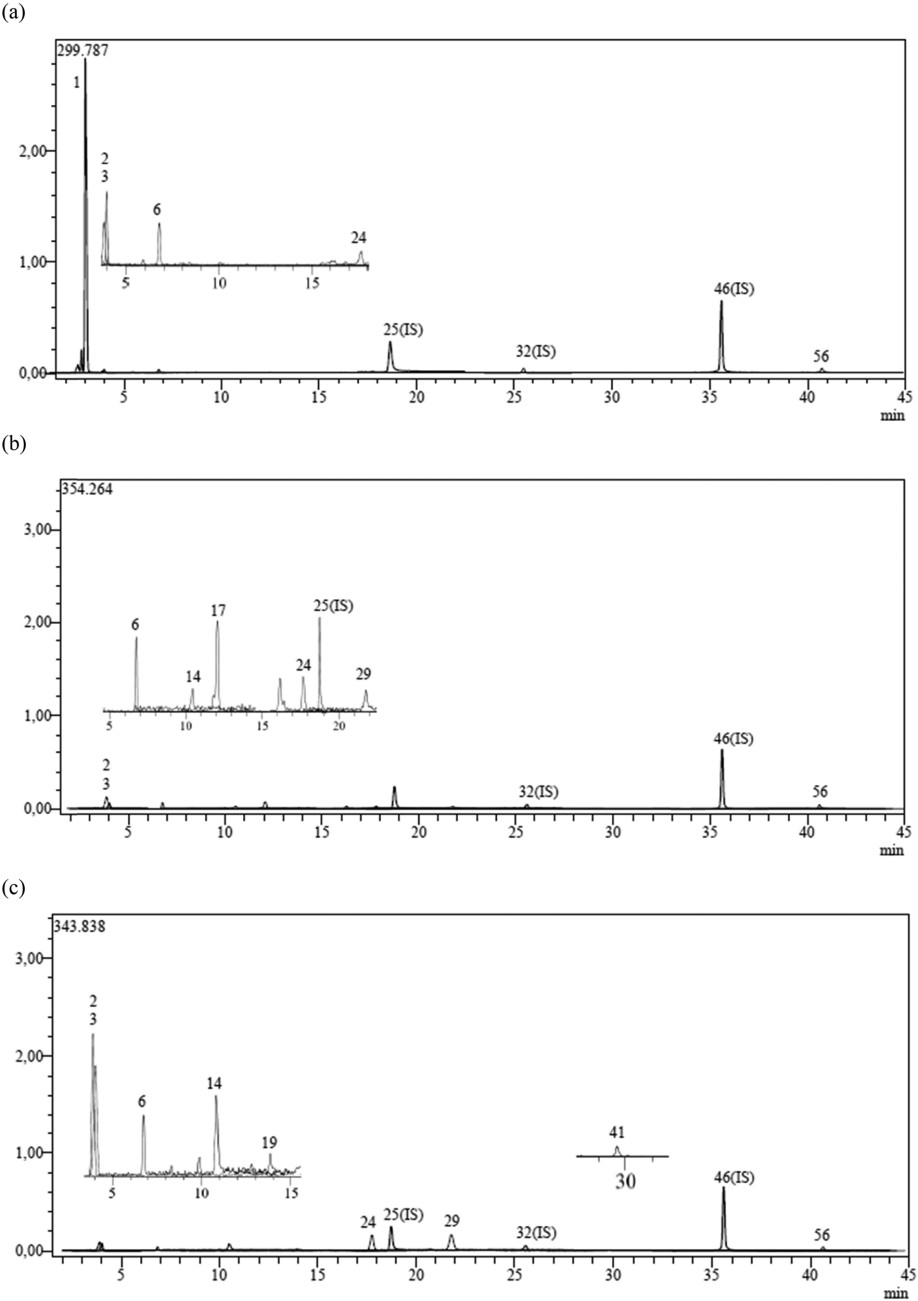
(a–c) Chromatograms of the cyclohexanolic fraction, butanolic fraction, and remaining AF of *C. ambrosioides*, respectively.

**Figure 2:**
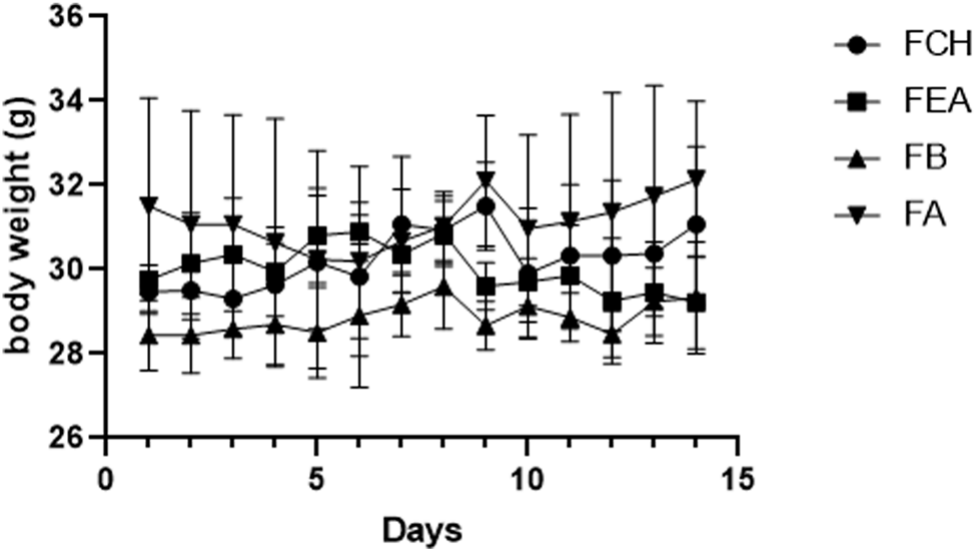
Body weight changes in the *Ammi majus* L. treated and control groups.

The chemical composition of the plant was determined using the LC–MS/MS method. The results of the identified compounds within the plant are presented in Table 1 and Figure 1. This table showcases the various chemical compounds present in the plant and their respective percentages in the overall composition (Figure 2).

**Table 1:** Compounds identified in extracts and fractions of *C. ambrosioides* via LC–MS/MS

Compound name	F_CH_	F_B_	F_A_
Fumaric acid	—	0.094	0.139
Aconitic acid	—	0.018	0.025
Protocatechuic acid	0.039	0.477	0.061
4-OH benzoic acid	—	—	—
Caffeic acid	—	0.014	—
*p*-Coumaric acid	—	0.219	0.037
Salicylic acid	—	0.076	—
Acacetin	0.036	0.03	0.023
Vanillin	0.114	—	
Quercitrin acid	—	—	2.934
Gallic acid	—	0.013	—
Gentisic acid	—	0.147	—
Chlorogenic acid	—	0.206	—
Rutin	—	4.1	—
isoquercitrin	—	0.182	—
Hesperidin	—	2.1	—
Quercitrin	—	0.039	—
Astragalin	—	0.059	—
Nicotiflorin	—	1.805	—
Quercetin	—	0.127	—
Kaempferol	—	0.019	—
Syringic aldehyde	0.059	—	—

We employed gas chromatography coupled with mass spectrometry (LC–MS/MS) to identify and measure compounds present in distinct fractions of *C. ambrosioides*. The outcomes of this analysis yield crucial insights into bioactive compounds potentially responsible for the plant’s medicinal properties.

The cyclohexanolic fraction (F_CH_) (chloroform) exhibited the presence of protocatechuic acid, vanillin, syringaldehyde, and acetin. These compounds have demonstrated various health benefits, such as antioxidant and anti-inflammatory effects. Their detection in the F_CH_ highlights its promise as a source of bioactive compounds warranting further investigation for medicinal applications.

In the butanolic fraction (F_B_), a diverse array of compounds was observed, including organic acids, flavonoids, and phenolic acids. Notably, rutin, hesperidin, and nicotiflorine, flavonoids recognized for their antioxidant properties, were detected in significant quantities, accentuating the potential health advantages of this fraction. Furthermore, the presence of quinic acid, gallic acid, and chlorogenic acid contributes to the antioxidant potential of the BF.

Remarkably, the aqueous fraction (F_A_) also contained compounds like quinic acid, fumaric acid, aconitic acid, and assorted phenolic compounds. This convergence of compounds among the fractions emphasizes the intricate nature of *C. ambrosioides* and emphasizes the necessity for further research to comprehend the synergistic interactions among these compounds.

In summary, LC–MS/MS analysis of fractions derived from *C. ambrosioides* unveiled a diverse and abundant chemical composition. The identification of organic acids, phenolic compounds, flavonoids, and fatty acids suggests the potential health and culinary benefits of this plant. These findings serve as a foundation for further investigation aimed at elucidating the specific bioactive properties and potential therapeutic applications of *C. ambrosioides*. Moreover, these results underscore the significance of exploring natural sources of bioactive compounds that can positively impact human health and overall well-being.

### 3.1 Acute toxicity

An acute toxicity study of fractions from *C. ambrosioides* was assessed in mice, following the OECD guidelines, code 423, for testing of chemical substances adopted in March 1996. The results of this investigation concerning the acute toxicity of orally administered plant extracts were encouraging. No deaths or clinical signs of toxicity were observed following administration of doses at 300 and 2,000 mg/kg of body weight. All animals survived the 14-day observation period, indicating that the LD50 (median lethal dose) is greater than 2,000 mg/kg. In accordance with the Globally Harmonized System of Classification and Labelling of Chemicals, the extracts can be considered non-toxic via oral administration.

Furthermore, monitoring the weight evolution of mice treated with fractions of *C. ambrosioides* during the observation period revealed notable stability in body weight, particularly after 14 days. This finding confirms that the fractions of *C. ambrosioides* have no toxic effects on the basic behavior of treated mice. These results are promising regarding the potential use of these extracts in various applications, emphasizing their safety when administered orally.

### 3.2 Subacute toxicity

#### 3.2.1 Animal body weight

Table 1 displays the variation in body weight of groups treated with fractions of *C. ambrosioides* at 200 mg/kg during the subacute toxicity study. This table is crucial in evaluating the impact of these substances on the subjects’ body weight. Here is an analysis of this data:

First, it is important to note that the table presents data for different weeks (weeks 1, 2, 3, and 4) and for different groups (control, F_CH_, F_EA_, F_B_, and F_A_). Each group appears to have been exposed to a dose of 200 mg/kg of *C. ambrosioides*, albeit in different fractions.

Upon examining the data, it is observed that the average body weight of mice in the control group was around 25.75 g in week 1 and slightly increased over subsequent weeks. Comparatively, the groups treated with fractions of *C. ambrosioides* also show an increase in body weight over the weeks. However, it is noteworthy that the variations between the different groups and weeks are relatively small.

Overall, these data indicate that exposure to 200 mg/kg of *C. ambrosioides* in fraction form did not result in significant variations in body weight compared to the control group. Furthermore, the minor fluctuation in body weight suggests that these substances did not have major toxic effects on the growth or development of mice during this subacute study period. This reinforces the notion that these fractions are well tolerated by mice at this dose and duration.

However, it is important to note that these variations in body weight are not necessarily indicative of toxicity. Other factors such as appetite, digestion, and metabolism can influence weight gain. Additionally, these results must be interpreted within the context of the study, considering other toxicity parameters such as biochemical and histopathological indicators (Table 2).

**Table 2:** Variation in body weight of groups treated with *C. ambrosioides* fractions at 200 mg/kg (g) in the subacute toxicity study

	Control	*F*_CH_	*F*_AE_	*F*_B_	*F*_A_
Week 1	25.75 ± 1.18	29.5 ± 1.04	30.11 ± 0.5	29.32 ± 0.7	27.01 ± 0.12
Week 2	26.43 ± 1.34	29.7 ± 1.05	30.72 ± 1.2	30.1 ± 1.5	27.91 ± 1.03
Week 3	27.43 ± 1.12	30.51 ± 0. 9	31.52 ± 0.9	30.9 ± 1.07	29.03 ± 1.13
Week 4	27.43 ± 0.56	31.33 ± 0.53	32.2 ± 1.2	31.22 ± 0.3	30.04 ± 0.11

Les valeurs sont exprimées en moyenne ± SD (*n* = 6).

### 3.3 Relative weight of organs

Table 3 displays the relative weights of vital organs for the treated and control groups at 200 mg/kg, providing further details on the toxicological effects of *C. ambrosioides* fractions.

**Table 3:** Relative weights of vital organs in treated and control groups in the subacute toxicity study of *C. ambrosioides* fractions

Organs	Control	*F*_CH_	*F*_AE_	*F*_B_	*F*_A_
Liver	1.63 ± 0.09	1.27 ± 0. 62	1.34 ± 0.2	1.6 ± 0.44	1.53 ± 0.19
Kidneys	0.37 ± 0.04	0.47 ± 0.06	0.4 ± 0.12	0.41 ± 0.16	0.4 ± 0.09
Lung	0.32 ± 0.09	0.29 ± 0.09	0.31 ± 0.03	0.3 ± 0.02	0.33 ± 0.05
Spleen	0.12 ± 0.01	0.23 ± 0.11	0.23 ± 0.05	0.24 ± 0.23	0.27 ± 0.28
Pancreas	0.09 ± 0.06	0.14 ± 0.05	0.13 ± 0.02	0.14 ± 0.04	0.14 ± 0.02

Values are expressed as mean ± SD (*n* = 6) and analyzed by one-way analysis of variance (ANOVA) followed by Tukey’s post hoc test.

Table 3 provides an overview of the variations in relative organ weights among different treatment groups and the control group. The examined organs include the liver, kidneys, lungs, heart, and pancreas. These organs are crucial for proper bodily functions, and any alteration in their weight can be an indicator of the potential toxicity of *C. ambrosioides* extracts.

Regarding the liver, the largest organ on the table, a slight decrease in relative weight is observed in the fraction A (*F*_AE_) and cyclohexane fraction (*F*_CH_) administration groups compared to the control group. This decrease may suggest some influence of the fractions on the liver, although the changes are not significant.

Concerning the kidneys, the relative weight remains relatively stable in all groups, indicating that *C. ambrosioides* fractions have no significant effect on this vital organ. For the lungs, there is also stability in relative weight across all groups, indicating an absence of notable effects on this organ.

The spleen, a central vital organ, shows more pronounced variations. The fractions seem to cause a slight increase in the spleen’s relative weight, which could indicate some influence on this organ. However, it is important to note that these variations remain within acceptable limits.

Regarding the pancreas, there is a slight increase in relative weight in all fraction groups, indicating some influence of the fractions on this organ. Overall, it appears that *C. ambrosioides* fractions have minimal effects on the vital organs examined in this subacute toxicity study. The variations in relative weight are generally slight and remain within acceptable limits. This suggests that the doses administered in this study did not result in major toxicity to these vital organs. However, it is important to note that toxicity may also depend on exposure time and doses administered.

The presented results are specific to a subacute study, and longer-term studies could reveal other potential effects. In conclusion, the results from this table indicate that *C. ambrosioides* fractions seem to have minimal impact on the vital organs studied in the context of this subacute toxicity study.

The results presented in Table 4 offer a more in-depth examination of the biochemical parameters in the control group and groups treated with fractions of *C. ambrosioides* at a dose of 200 mg/kg.

**Table 4:** Biochemical parameters of the control group and groups treated with *C. ambrosioides* fractions at 200 mg/kg for subacute toxicity study

Parameter	Control	*F*_CH_	*F*_AE_	*F*_B_	*F*_A_
AST (U/dL)	48.40 ± 6.21	50.05 ± 5.12	48.79 ± 5.44	46.53 ± 7.32	49.13 ± 6.01
ALT (U/dL)	103.20 ± 8.7	104. 77 ± 3.23	105.9 ± 3.92	106.21 ± 5.6	105.21 ± 1.8
CR (mg/dL)	1.06 ± 0.30	1.55 ± 2.77	1.44 ± 0.5	1.55 ± 0.38	1.66 ± 0.41
UR (g/dL)	0.59 ± 0.07	0.52 ± 0.2	0.6 ± 0.1	0.56 ± 0.03	0.53 ± 0.1
TP (g/dL)	62.88 ± 0.22	58. 58 ± 4.54	60.72 ± 6.54	65.48 ± 5.2	62.33 ± 4.06
TC (mg/dL)	127.6 ± 8.70	129.83 ± 8.99	124.05 ± 5.15	125.36 ± 6.9	126.06 ± 7.1
HDL (mg/dL)	7.36 ± 0.06	7.23 ± 0.54	7.28 ± 1.61	6.54 ± 0. 59	6.9 ± 1. 09
LDL (mg/dL)	60.15 ± 1.01	61.2 ± 5.43	61.31 ± 3.5	62.03 ± 4.23	63.23 ± 3.14
VLDL (mg/dL)	26.26 ± 0.15	28.36 ± 0.98	25. 81 ± 2.03	26.07 ± 0.34	27.23 ± 1.12

### 3.4 AST and ALT – liver enzymes

The liver is a multifunctional organ responsible for various biochemical processes in the body, and enzymes like AST and ALT are crucial indicators of liver health. The results reveal an intriguing trend in the levels of these enzymes among the treatment groups.

AST and ALT: AST and ALT are important liver enzymes. The table shows significant reductions (*P* < 0.05) in AST and ALT levels in the treated groups (*F*_CH_, *F*_AE_, *F*_B_, *F*_A_) compared to the control group. These results suggest that these extracts do not affect liver function, indicating a potential reduction in hepatic toxicity.

However, it is essential to emphasize the need for further in-depth statistical analysis to confirm the significance of these results and elucidate the exact mechanisms underlying this hepatoprotective effect.

### 3.5 CR and UR – markers of renal function

CR and UR are commonly used as markers to assess renal 10 function. Regarding urea (UR) levels, which are indicators of kidney function, no significant difference was observed between the control group and the treated groups. This indicates that the extracts and fractions did not negatively affect kidney function.

Indeed, it should be noted that there was a consistent increase in CR levels in all groups treated with the different extracts and fractions of *Chenopodium ambrosioides*. This elevation in CR levels may indicate a potential effect on kidney function associated with the administration of these substances. Although the precise mechanisms underlying this observation need to be elucidated, such consistent alterations in creatinine levels across multiple treatment groups warrant careful examination.

### 3.6 Total proteins (TP)

TP are crucial constituents of blood and play an essential role in maintaining various physiological functions. In this study, group EI shows a substantial increase in TP levels compared to the control group, and this increase is statistically significant (*P* < 0.05). Conversely, the *F*_CH_ group presents a statistically significant decrease (*P* < 0.05) in TP levels.

These results deserve further investigation to understand the factors influencing these variations. The significant decrease in TP observed in *F*_CH_ could be due to reduced protein synthesis or increased protein degradation.

The changes in TP levels are intriguing and underline the need for continued research on the mechanisms responsible for these alterations. Understanding the underlying reasons for these variations can provide valuable insights into the potential impact of treatments on protein metabolism.

### 3.7 Total cholesterol (TC), high-density lipoprotein (HDL), LDL, and very-low-density lipoproteins (VLDL) – cholesterol profile

Cholesterol is a vital biomarker in evaluating cardiovascular health. TC levels in the treatment groups do not significantly differ (*P* < 0.05) from those in the control group. It is noteworthy that TC levels in all groups fall within the typical physiological range. This suggests that the treatments, although not inducing substantial changes, did not lead to cholesterol levels considered abnormal.

LDL levels show variations, but there is no significant trend. This suggests that these fractions do not have a major effect on lipoproteins associated with cardiovascular diseases. However, it is essential to emphasize that levels of HDL, often referred to as “good” cholesterol, remain relatively stable in all groups without statistically significant variations. This stability in HDL levels can be considered positive for cardiovascular health.

Additionally, VLDL are another marker associated with cardiovascular health. It is crucial to note that values across all groups remain within the normal range, indicating that the treatments did not induce adverse effects related to cholesterol levels.

## 4 Discussion

*C. ambrosioides*, a member of the Chenopodiaceae family, has sparked keen interest due to its potential medicinal properties. This plant is traditionally known for its use in various herbal remedies. It is essential to assess its safety profile, encompassing evaluations of both acute and subacute toxicity, to understand its viability for therapeutic applications.

First, it is noteworthy that the fractions exhibit a diverse array of compounds, ranging from organic acids to flavonoids and phenolic acids. This diversity suggests the complexity of the plant’s chemical profile and hints at the multifaceted nature of its potential medicinal properties.

One compound of interest is fumaric acid, which is detected in both the F_B_ and F_A_ but not in the F_CH_. Fumaric acid is known for its antioxidant properties and potential therapeutic effects, particularly in the treatment of psoriasis. Its presence in the fractions indicates the potential contribution of *C. ambrosioides* to antioxidant activity.

Another notable finding is the presence of various phenolic acids, such as caffeic acid, *p*-coumaric acid, and gallic acid, across different fractions. Phenolic acids are renowned for their antioxidant, anti-inflammatory, and anticancer properties. Their presence underscores the potential health benefits associated with *C. ambrosioides* consumption.

Furthermore, flavonoids like rutin, hesperidin, and quercetin are detected in the fractions, particularly in the BF and AF. Flavonoids are well known for their diverse pharmacological activities, including antioxidant, anti-inflammatory, antiviral, and anticancer effects. The abundance of flavonoids in the fractions suggests that *C. ambrosioides* may possess significant therapeutic potential.

Additionally, the presence of other bioactive compounds such as acacetin, nicotiflorin, and quercitrin acid further enriches the chemical profile of the fractions. These compounds have been associated with various health benefits, ranging from cardiovascular protection to anti-inflammatory effects.

The results from the acute toxicity assessment provided encouraging information regarding the safety of *C. ambrosioides* fractions. No deaths or signs of toxicity were observed at doses up to 2,000 mg/kg, indicating that the LD50 is greater than 2,000 mg/kg. This finding is significant, especially considering the potential therapeutic applications of this plant.

In accordance with OECD guidelines, the plant fractions are considered non-toxic when administered orally. This aligns with the expectation that many plant fractions can be well-tolerated at moderate to high doses. Such findings can reinforce the use of *C. ambrosioides* in various therapeutic applications, ranging from traditional medicine to modern pharmacopeia.

However, it is important to remember that acute toxicity assessments have limitations. They provide valuable initial indications of a substance’s safety but do not integrate potential long-term or cumulative effects.

Subacute toxicity studies offer a more detailed perspective on the effects of *C. ambrosioides* fractions, especially when administered over an extended period. These studies evaluated various parameters, including body weight, relative organ weight, and biochemical parameters, to obtain a comprehensive understanding of the plant’s impact.

Maintaining stable body weight within treated groups over a 4-week period suggests that the fractions, at a dose of 200 mg/kg, did not significantly affect overall health and development in mice. These results are reassuring for subacute applications.

When examining relative organ weights, no significant differences were observed in vital organs such as the liver, kidneys, lungs, spleen, and pancreas. Slight variations were noted but were not considered significant. These results indicate that *C. ambrosioides* extracts have minimal effects on the relative weight of these crucial organs, further reinforcing their safety.

The biochemical evaluations of the subacute toxicity study explored liver and kidney function, as well as lipid profiles. The results of AST and ALT levels, which are liver enzymes, are particularly intriguing. The groups receiving the extracts showed reduced levels of AST and ALT, with the reduction being statistically significant. This suggests that the fractions may have hepatoprotective properties, potentially attenuating liver damage or stress. The potential hepatoprotective effect of these fractions is a significant discovery and may pave the way for further research into their application in liver-related conditions.

However, it is essential to recognize that the reduction in AST and ALT levels, while promising, requires further investigations to establish the precise mechanisms involved and their clinical implications. Moreover, while the results of this study suggest hepatoprotection, they do not entirely eliminate the need for comprehensive safety evaluations, especially in scenarios of long-term or chronic exposure.

The subacute toxicity study also evaluated parameters related to kidney function, such as creatinine (CR) and urea (UR) levels. We observed a consistent increase in creatinine levels in all treated groups, suggesting a potential influence on kidney function. This raises concerns about the safety of Chenopodium ambrosioides extracts and fractions regarding their long-term effects on kidney function.

The results related to lipid profiles raise important questions. Although the levels remain within the normal range, these results highlight the importance of in-depth research into the cardiovascular implications of *C. ambrosioides* fractions.

These findings open new perspectives in the field of medicinal plant research. *C. ambrosioides*, with its promising safety profile and hepatoprotective properties, offers potential for various therapeutic applications. However, caution is warranted as further research is essential to unlock its full therapeutic potential and understand the long-term implications. The safety of natural remedies, such as fractions of *C. ambrosioides*, must be rigorously evaluated, considering the growing interest in herbal medicine and natural products.

The essential oil of *C. ambrosioides* at a concentration higher than 35 μg/mL has shown cytotoxicity and neurotoxicity towards G-749 cells, normal human fibroblasts, with an IC50 of 207.1 ± 4.4 μg/mL (Soares et al.) [18]. Meanwhile, a study conducted by Buckle [19] revealed that *C. ambrosioides* itself is neurotoxic with a narrow therapeutic range; the toxicity was attributed to the presence of camphor and ascaridole. Another study by Monzote et al. [20] found that the toxic effects of caryophyllene oxide and carvacrol present in *C. ambrosioides* may be mediated by the inhibition of complex I in mitochondrial electron transport chains, while the toxicity of ascaridole towards mammalian mitochondrial oxidative phosphorylation depends on the presence of ferrous iron (Fe^2+^).

## 5 Conclusion

Some botanical substances may demonstrate systemic toxicity with prolonged exposure, including reduced body mass, altered behavior, and hematological and biochemical changes. These risks often arise from plant components inducing hepatic and renal toxicity. In our investigation, fractions extracted from *C. ambrosioides* using increasingly polar solvents aimed to pinpoint specific compounds responsible for potential toxicity. Remarkably, the acute toxicity study at a dosage of 2,000 mg/kg showed no mortality among treated mice. However, marginal shifts in organ weight observed in the subacute toxicity study at 200 mg/kg were deemed non-toxic. Notably, no macroscopic or microscopic alterations were detected in the organs, and AST levels remained unchanged, indicating an absence of hepatic toxicity. Furthermore, the treatment did not affect cholesterol levels, suggesting an intact lipid balance in the mice.

Subacute treatment with these fractions resulted in minimal deviations within the treated groups. These slight changes may be associated with disruptions in kidney and liver function. However, observed alterations in the context of the subacute study do not constitute toxic effects. This investigation highlights that despite minor variations, *C. ambrosioides* fractions exhibit a relatively benign impact, without inducing severe or demonstrable toxicity within the observed parameters. These findings contribute to understanding the safety profile of *C. ambrosioides* fractions, advocating for a cautious approach due to potential associations with kidney and liver function, albeit without significant pathological changes.

Future studies should focus on elucidating the specific mechanisms underlying the observed effects and exploring the potential therapeutic advantages of natural plant extracts from *C. ambrosioides*. Understanding these mechanisms can inform the development of safer and more effective botanical-based treatments.
